# Statistically Optimized Production of Saccharides Stabilized Silver Nanoparticles Using Liquid–Plasma Reduction Approach for Antibacterial Treatment of Water

**DOI:** 10.3390/ma14195841

**Published:** 2021-10-06

**Authors:** Noor Ul Huda Altaf, Muhammad Yasin Naz, Shazia Shukrullah, Haq Nawaz Bhatti, Muhammad Irfan, Mabkhoot A. Alsaiari, Saifur Rahman, Usama Muhammad Niazi, Adam Glowacz, Klaudia Proniewska, Lukasz Wzorek

**Affiliations:** 1 Department of Physics, University of Agriculture Faisalabad, Faisalabad 38040, Pakistan; noorulhuda100@yahoo.com; 2 Department of Chemistry, University of Agriculture Faisalabad, Faisalabad 38040, Pakistan; haq_nawaz@uaf.edu.pk; 3 Electrical Engineering Department, College of Engineering, Najran University Saudi Arabia, Najran 11001, Saudi Arabia; miditta@nu.edu.sa (M.I.); srrahman@nu.edu.sa (S.R.); 4 Empty Qaurter Research Unit, Chemistry Department, College of Science and Art at Sharurah, Najran University Saudi Arabia, Najran 61441, Saudi Arabia; mabkhoot.alsaiari@gmail.com; 5 Department of Mechanical Engineering Technology, National Skills University, Islamabad 44000, Pakistan; ukniaxi@gmail.com; 6 Department of Automatic Control and Robotics, Faculty of Electrical Engineering, Automatics, AGH University of Science and Technology, al. A. Mickiewicza 30, 30-059 Krakow, Poland; adglow@agh.edu.pl; 7 Department of Bioinformatics and Telemedicine, Jagiellonian University Medical College, Anny 12, 31-008 Krakow, Poland; klaudia.proniewska@uj.edu.pl; 8 Wzorek Systems, ul. Kapelanka 10/18, 30-347 Krakow; Poland; lukasz@wzorek.systems

**Keywords:** silver nanoparticles, plasma electrolysis, mono- and poly-saccharides, response surface methodology, antibacterial activity

## Abstract

Various conventional approaches have been reported for the synthesis of nanomaterials without optimizing the role of synthesis parameters. The unoptimized studies not only raise the process cost but also complicate the physicochemical characteristics of the nanostructures. The liquid–plasma reduction with optimized synthesis parameters is an environmentally friendly and low-cost technique for the synthesis of a range of nanomaterials. This work is focused on the statistically optimized production of silver nanoparticles (AgNPs) by using a liquid–plasma reduction process sustained with an argon plasma jet. A simplex centroid design (SCD) was made in Minitab statistical package to optimize the combined effect of stabilizers on the structural growth and UV absorbance of AgNPs. Different combinations of glucose, fructose, sucrose and lactose stabilizers were tested at five different levels (−2, −1, 0, 1, 2) in SCD. The effect of individual and mixed stabilizers on AgNPs growth parameters was assumed significant when p-value in SCD is less than 0.05. A surface plasmon resonance band was fixed at 302 nm after SCD optimization of UV results. A bond stretching at 1633 cm^−1^ in FTIR spectra was assigned to C=O, which slightly shifts towards a larger wavelength in the presence of saccharides in the solution. The presence of FCC structured AgNPs with an average size of 15 nm was confirmed from XRD and EDX spectra under optimized conditions. The antibacterial activity of these nanoparticles was checked against Staphylococcus aureus and Escherichia coli strains by adopting the shake flask method. The antibacterial study revealed the slightly better performance of AgNPs against Staph. aureus strain than *Escherichia coli*.

## 1. Introduction

Silver nanoparticles (AgNPs) are being used as an antimicrobial agent in water treatment [1], cosmetics, animal feed wounds and burn treatment [2], remediation of bacteria and other micro-organisms on fabrics surfaces [3], inhibition of bacterial biofilm on catheters [4], and so on. Particularly, AgNPs with high ratio of surface area to volume (size below 100 nm) are considered suitable for antimicrobial applications to neutralize the growth of gram-positive and gram-negative strains. These nanoparticles also find their applications in cosmetics, cancer treatment, tissue scaffolds as food additives, textile fabrics, pharmaceutics, etc. [5].

Various methodologies have been reported in the literature to synthesize metal nanoparticles, including hydrothermal method [6], sol-gel method [7], photodetection technique [8], ultraviolet irradiation method [9], electrochemical technique [10], porous materials template [11], solvothermal method [12], chemical synthesis [13], microwave plasma treatment [14], polyol process [15], green synthesis [16,17] coaxial electro-spinning [18] and liquid plasma interaction technique [19]. Each approach has its own set of advantages and disadvantages, including differences in process cost, quantity, functionality and particle size distribution. The liquid–plasma interaction (LPI) method is thought to be simple, rapid and cost-effective. This technology can produce NPs in a safe environment without the use of harmful chemicals. The production of nanoparticles through conventional physical and chemical methods involves the reduction of metal salts into nanoparticles with suitable reducing agents and stabilizers [20,21]. The LPI is a suitable method to synthesize nanoparticles of specific sizes and shapes. The increased surface energy of nanoparticles results in particle aggregation by effecting the surface area, reactivity and antibacterial applications [22]. As a result, the aggregation effect is the most important consideration for effective AgNP applications and colloidal stability. It is important to minimize the aggregation for effective therapeutic and antibacterial applications as lower stability leads to lower antibacterial performance [20,23]. Different stabilizing agents such as polyvinylpyrrolidone (PVP) [24], HCl, HNO_3_ [25], sodium dodecyl sulfate (SDS) [26], polymers [27], etc., have been utilized for AgNPs synthesis. These stabilizing agents are costly and potentially hazardous to our ecosystem and cause tremendous biological risks. Thus, monosaccharides- and polysaccharides-assisted synthesis of AgNPs is suggested as a cheap and eco-friendly approach, which further consolidates their position as one of the best choices for sustainable synthesis of nanoparticles. 

It is important to note that all studies that reported on the effect of stabilizing agents for the production of AgNPs are single variable experiments. Single variable optimization procedures are not only time consuming, costly and labor intensive, but they can also lead to a misinterpretation of results, particularly when there are substantial interactions between the parameters being investigated [28]. As a result, procedures for factor optimization using multivariate techniques including Box–Behnken design, mixture design, central composite design and simplex centroid design (SCD) are preferred since these procedures are suitable, faster, cost-effective and allow for simultaneous optimization of multiple variables [29]. This paper presents a simplex centroid experimental scheme to establish a relationship among response and input variables. It allows us to estimate the interaction of each element in the mixture by providing a component that represents the interaction and optimizing the component elements according to the targets. It not only creates a surface model of continuous variables by estimating each element and their interactions in the mixture, but it also optimizes the elemental components to study the best combined effect of mixture elements [30].

The effect of different combinations of stabilizers on LPI is not reported well in the literature. In addition, statistical optimization of the process parameters for the liquid–plasma interaction method has never been performed. The nanomaterials have shown low performance in many applications due to unoptimized synthesis conditions and structural parameters. The unoptimized studies not only raise the process cost but also complicate the physicochemical characteristics of the nanostructures. In this study, an argon plasma jet was used to reduce the solution of metal salts and stabilizers for controlled synthesis of AgNPs. The synthesis parameters were optimized statistically and experimentally. Nanoparticles of Ag were synthesized by the liquid–plasma interaction method. Glucose, fructose, sucrose and lactose stabilizers were used as synthesis parameter for controlled synthesis of Ag under argon plasma jet exposure. Optimal synthesis conditions were obtained by implementing SCD mixture design. The prepared AgNPs were tested for their antibacterial performance against Staph. aureus strain and *E. coli* strains. This research contributes new information on the optimal interaction effect of stabilizers on the structural development and UV absorbance of AgNPs, which has previously been described using other comparable methodologies [31,32].

## 2. Experimental Section

### 2.1. Materials

AgNO_3_, glucose, fructose (C_6_H_12_O_6_), sucrose and lactose (C_12_H_22_O_11_) were supplied by Sigma Aldrich (St. Louis, MO, USA). Deionized water was utilized for the preparation and washing of NPs.

### 2.2. Synthesis of Silver Nanoparticles

**Figure 1:**
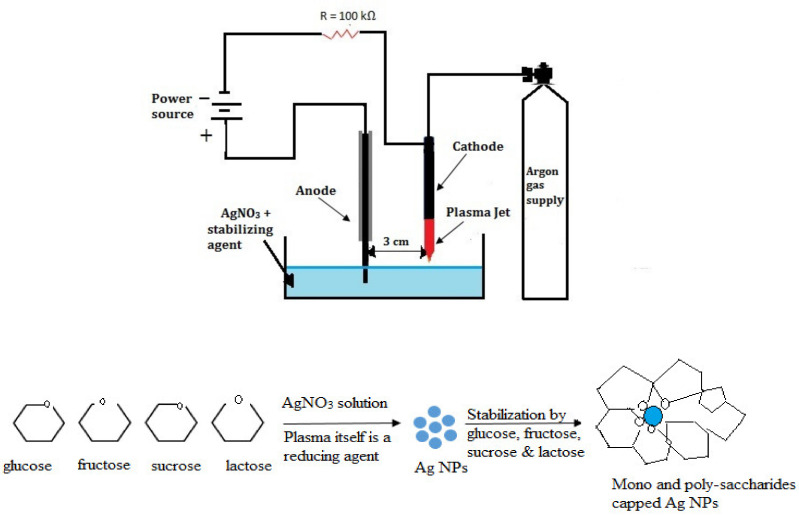
Illustration of experimental setup for liquid-plasma reduction, proposed mechanism and graphical representation for the formation of AgNPs.

Experimental setup and associated mechanism of liquid–plasma setup for synthesis of nanoparticles is illustrated in Figure 1. A similar setup was used in our previously reported work [32]. A 20 kV direct current supply was used to create argon plasma stream by flowing argon gas through a 2 mm hollow cathode. When the applied voltage was 10 kV and the current through the circuit was 40 mA, a sustained plasma stream was formed at an argon flow rate of 1200 sccm. The level of input voltage and current flowing through the circuit is affected by the separation between the cathode and the positively charged solution, as well as the solution’s conductivity. In this work, the plasma stream on interaction with solution supplies reactive species to initiate the reduction of silver ions to nanoparticles. The saccharides in an aqueous solution occupy 40% of total aqueous volume and impose physical constraints that separates AgNPs from one another to prevent the aggregation effect.

About 100 mL electrolyte solution of silver nitrate (AgNO_3_) and deionized water was prepared with molar concentration of 5 mM for 60%. The monosaccharides, namely glucose, fructose, sucrose and lactose with concentration of 2 mM for 40% were used to stabilize the growth process. Stoichiometric amount of DI water was mixed with silver nitrate solution to produce reaction volume of 100 mL. After that, the silver anode was immersed in the aqueous solution. The upper part of anode was covered with a quartz-glass tube. The gas nozzle was used as cathode, which was connected with a gas cylinder. The plasma treatment dissociates the electrolyte solution of AgNO_3_ into Ag^+^ cations and NO_3_^−^ anions as:

(1) $${\;\mathrm{AgNO}}_{3}\to {\mathrm{Ag}}^{+}+{\;\mathrm{NO}}^{3-}$$

To avoid the electrode burning, a graphite electrode of 5 mm thickness was used as an anode. By applying DC voltage between anode and metallic nozzle, DC discharge plasma was generated under argon flow. The applied voltage may vary from 0–20 kV and discharge current from 1–50 mA. The interaction time between the plasma and the solution is set to 30 min. Thirty experiments with different molar concentrations of monosaccharide sugars were carried out to investigate the effect of sugar molar concentration on particle size and dispersion of AgNPs. The energetic electrons and radicals, generated by plasma discharge, reduce Ag^+^ cations into AgNPs ($\mathrm{Ag}+e^{-}\to Ag$). The transparent color of the solution changes to black over time, which indicates the formation of AgNPs.

### 2.3. Experimental Scheme in Simplex Centroid Design

A simplex centroid design (SCD) was used to obtain the optimum combinations of glucose (A), fructose (B), sucrose (C) and lactose (D) stabilizers for the synthesis of AgNPs. The variables were taken in the concentration of 40% to test at five different levels (–2, –1, 0, 1, 2). 5 mM AgNO_3_ 60% (*v*/*v*) was chosen as the center value (zero level) in the experimental design. The design comprising 30 assays was implemented systematically, as shown in Table 1. The experiments displayed in mixture design were executed in 100 mL electrolyte solution containing AgNO_3_ and saccharides. By using SCD, all the experiments were optimized by a UV–Vis spectrophotometer, and the absorbance response of every trail at 302 nm was recorded. The response surface methodology (RSM) was employed to assess the results of SCD experiments using a 2nd order polynomial equation, given as [33,34]:

AgNPs (Y) = β_0_+ Σβ_i_X_i_ + Σβ_ii_X_i_^2^ + Σβ_ij_X_i_X_j_ (2)

where Y represents predicted response variable and β_0_ and β_i_ represent the regression and linear coefficients, respectively. Similarly, β_ii_ and β_ij_ represent quadratic interaction coefficients, respectively. The coded levels of independent variables are represented by X_i_ (i = 1, 2, 3, 4, etc.). The independent variables were coded as: A, B, C and D.

(3) Y=β0+β1A+β2B+β3C+β4D+β12AB+β13AC+β14AD+β23BC+β24BD+β11A2+β22B2+β33C2+β44D22

The data were expressed in three-dimensional graphs to depict the unique and interaction effects of these investigative variables on the response. F-values, interaction plots, lack of fit, correlation between predicted and experimental values and R^2^ values were used to check the validity of the design. Finally, the analysis of variance was used to reflect the entire quadratic model (ANOVA).

### 2.4. Statistical Analysis

All trials were conducted in five different levels to ensure the correctness and rationality of the applied SCD design of RSM, as well as to interpret the response variable. ANOVA (analysis of variance) was performed by using the Minitab software. Multiple linear regressions were applied on the experimental data to estimate the interaction effects, sum of squares (SS), *p*-value, *t*-test and confidence level. The significance of regression coefficient parameter was checked through *t*-tests. The *p*-values were utilized to study how interaction effects significance, which may elaborate the interaction patterns between the variables [35]. The coefficient of correlation (R), the determination coefficient (R^2^) and the adjusted R^2^ values were used to indicate the regression model fit quality, and a statistics test was used to determine its statistical significance. RSM is an arithmetical model which is modified extensively and has been utilized for the optimization of several factors with no process variables. It describes the relation between the response and levels of the factors simultaneously by using statistical software. 

### 2.5. Antibacterial Activity

The antibacterial effect of the synthesized AgNPs was tested against model gram-positive and gram-negative bacteria strains *Staph aureus* and *E. coli* (*S. aureus*, *E-coli*). The composition of bacterial culture was made with 28 g of nutrient agar dissolved in 1000 mL of deionized water. The petri plates and borer were autoclaved for 15 min. Wells were prepared properly at the same distance after cooling down of autoclave media and pure bacteria beside the petri plates. 100 µL of the prepared sample was added to various plates. In brief, a bacterial suspension of 100 μL was spread uniformly on the surface of a nutrient agar plates. Then, filter paper disks (about 6 mm in diameter) impregnated with stabilized AgNPs were placed on the agar surface. The incubation of plates was conducted at 37 °C for 24 h after which the average diameter of the inhibition zone surrounding the disk was measured. Ciprofloxacin was used as control medicine to examine the antibacterial performance of the stabilized AgNPs.

## 3. Results and Discussions

### 3.1. Optimization of Synthesis Process Using Response Surface Methodology

Thirty tests in total with different combinations of stabilizing agents (A, B, C and D = 40%) were performed in this study, with 5-different levels of AgNO_3_ concentration (60%). Results of study are shown in a randomized manner in Table 2. The better production of AgNPs (>1.4) has been achieved by the interaction of pure and mixture of stabilizers in treatment runs one, three and sixteen. Under the conditions of 5 mM AgNO_3_ (60%, *v*/*v*), the maximal AgNPs synthesis (1.626) was observed in run number 16. The response and independent variables were tested to fit into a linear and quadratic equations and their second-order interactions were tested by multiple regression analysis on experimental data from SCD. The determination coefficient R^2^ and the F test ANOVA were performed to check the model quality.

The variations in predicted and real response and fit statistics were confirmed by determining coefficient (R^2^). The R^2^ number is always in the range of 0 to 1. The model’s R^2^ was 0.9657, indicating that the model could explain 96.57% of the variability in the response. A regression model with an R^2^-value greater than 0.9 is considered to have a high level of correlation [36]. The present R^2^-value indicates that the observed and expected responses are extremely well aligned, demonstrating that the model is reliable for AgNPs. Furthermore, the adjusted coefficient (Adj. R^2^ = 0.9338) is quite high, implying that the response functions are well-suited to the experimental data [37]. The synthesis of AgNPs peaked after 30 min of plasma exposure, and the predicted model had a high coefficient of determination (R^2^ = 0.9827) [38]. The results of fitting the second order response surface model with the analysis of variance are shown in Table 2. The significance and efficiency of the model must be tested using ANOVA.

The F-value, also known as the Fisher variance ratio, is a statistically valid measure of how well variables represent variation in data around their mean. The regression model was found to be significant at *p*-values less than 0.05 using analysis of variance (ANOVA). Fisher’s statistical analysis proved the adequacy of the developed model. All model coefficient values were determined using multiple regression analysis. The Student’s *t*-test and *p*-values were utilized to establish the coefficient significance, as shown in Table 3. The *p*-values were also tested to verify the coefficients’ significance, which are required to comprehend the mutual interaction pattern between the test variables. Interaction of two factors can have an antagonistic or synergistic effect.

**Figure 2:**
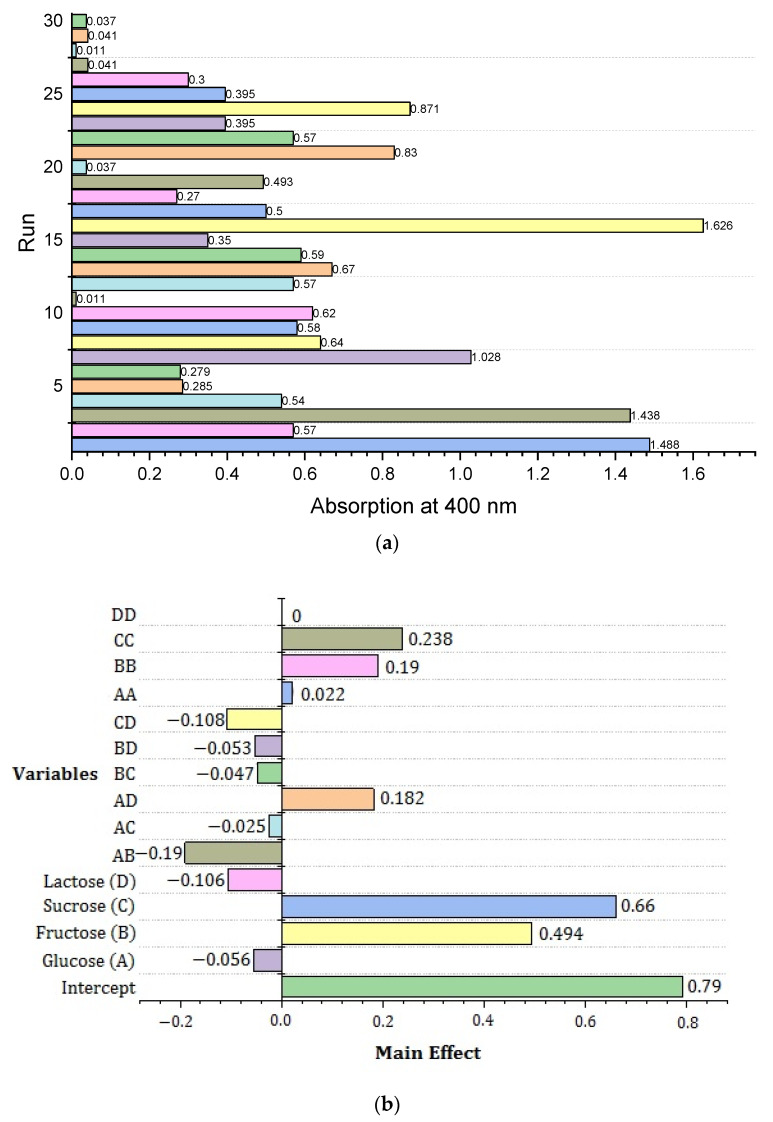
(**a**) Pareto chart illustration of assays of experiments with response variable. (**b**) The significance of the variables affecting AgNPs is represented by a Pareto chart, which shows positive and negative effects.

The quadratic B (fructose) and C (sucrose), and linear impacts of B (fructose), C (sucrose) and D (lactose), both significantly impact the synthesis of AgNPs. The variables A (glucose) and B (fructose) have the greatest interaction (0.003), indicating that they affect 99.657% of the model; followed by A (glucose) and D (lactose) (0.005), and then C and D. Interactions between A (glucose) and C (sucrose), interaction between B (fructose) and D (lactose), interaction between B (fructose) and D (lactose), as well as the quadratic effects of D and the linear effects of A did not have a significant impact on plasma synthesis of AgNPs. Created on second order response surface design, the interaction between optimum levels of glucose concentration (A), fructose (B), sucrose (C), lactose (D) and the response was revealed in the system of three-dimensional surface plots. The second-order response (Equation (4)) that characterizes the expected response (Y) in terms of the independent variables (A, B, C and D) was obtained using multiple regression analysis on experimental data:

(Y) Yield (AgNPs) = 0.395 − 0.028A + 0.247B + 0.330C − 0.053D − 0.095AB − 0.013AC + 0.091AD − 0.024BC − 0.026BD − 0.054CD + 0.011A^2^ + 0.095B^2^ + 0.119C^2^ + 0.000D^2^ (4)

where Y is the predicted response, A is the glucose coded value, B is the fructose coded value, C is the sucrose coded value and D is the lactose stabilizing agent coded value. A significant interactive effect was depicted in the Pareto chart, as shown in Figure 2a,b. Pareto analysis is a formal technique that ranks the influence of individual and interaction effects of the variables on response [38]. Among four components, the linear effects of intercept exhibited the highest positive significance (0.790). In addition to sucrose’s linear effect, fructose’s linear effect and sucrose’s quadratic effect had positive effects of 0.660 and 0.494. The negative effect of the interaction between glucose and the sucrose stabilizing agent was −0.025. Typically, the fitted model must be validated to ensure that it provides a good approximation to the real system. Analyzing and optimizing the fitted response surface unless the model suggests a satisfactory fit is likely to produce poor or misleading results. The literature suggests that the significance of each co-efficient is checked by noting the *p*-value [39]. The *p*-value also predicts the effect of different parameters on the response. The value less than 0.05 shows high significance of the corresponding co-efficient.

**Figure 3:**
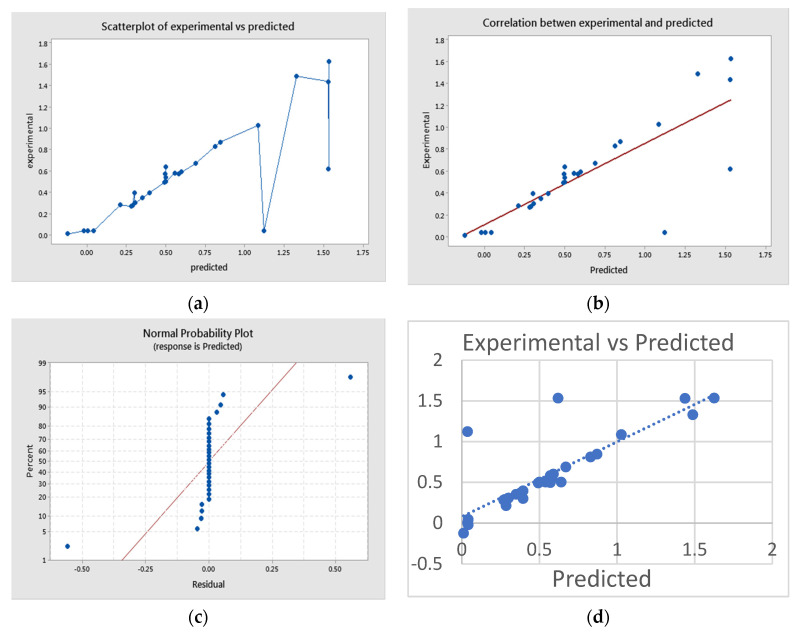
(**a**) Scatter plot of experimental versus predicted, (**b**) correlation between actual values and predicted values, (**c**) normal plot of residuals versus predicted values and (**d**) normal plot of predicted versus experimental values.

**Figure 4:**
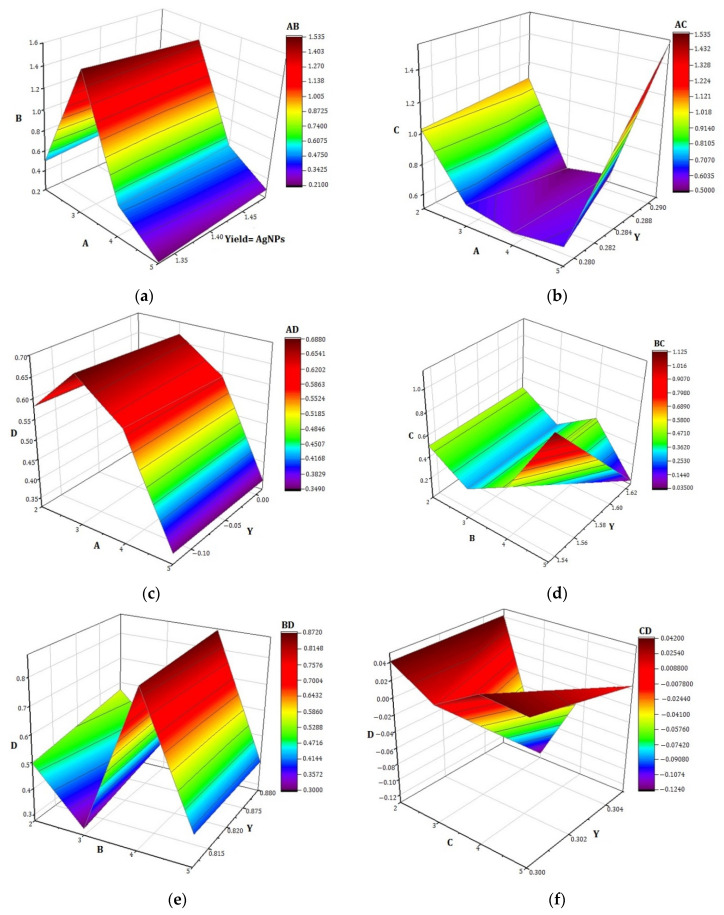
RSM surface plots (**a**–**f**) of the synthesized AgNPs revealing the effect of independent variables and their combination: AB, AC, AD, BC, BD, CD.

Figure 3a depicts the experimental V/S predicted scatter plot. The residuals were compared to the model’s predicted normal values. The points near the diagonal line in the normal probability plot of the residuals imply that the errors are normally distributed, independent of each other, and that the error variances are homogeneous. It predicts that model fits well for the experimental data. All of the model’s primary assumptions have been validated because the residuals of the fitted model are distributed normally. Figure 3b shows a plot between predicted and observed responses, demonstrating a good match between predicted values and experimental data. The model’s good fit is indicated by the points clustered around the diagonal line. The residual plot shown in Figure 3c reveals an equal scattering of residual data both below and above the *x*-axis, which indicates that variance is independent of formation of nanoparticles and that the model fit was satisfactory. Figure 3d shows a plot of experimental v/s predicted values for response variable as a visual diagnostic plot, indicating that the theoretical values predicted by the model equation are in close agreement, confirming the model’s adequacy. When one variable is set at the optimum value by allowing the other two variables to vary, the plot of response surface curves in Figure 4a–f shows optimal levels of the variables and interaction effects. 

The interaction of glucose and fructose stabilizing agents is depicted in Figure 4a. It showed that high fructose levels resulted in the highest yield of AgNPs. Lower glucose concentration sustains high levels of AgNPs yield, while higher glucose concentration allows AgNPs through the liquid–plasma interaction method to gradually decrease. Higher levels of sucrose support a significant production of AgNPs, as shown in Figure 4b. At a low concentration of glucose, the growth of AgNPs increases. As the concentration of glucose increased, the growth of AgNPs decreased. The interaction of glucose and lactose stabilizing agents is depicted in Figure 4c. It shows that a lower level of lactose and glucose quantity is supportive to the high production of AgNPs, but maximum lactose or glucose concentration led to a decrease in AgNPs synthesis. The three-dimensional plot of AgNPs as a function of fructose and sucrose stabilizing agents is shown in Figure 4d. It shows that increasing the concentration of fructose and sucrose stabilizing agents causes a progressive increase in AgNPs yield by the liquid–plasma interaction technique. The high level of fructose and sucrose produces the maximum yield of AgNPs. The interaction of fructose and lactose stabilizing agents is depicted in Figure 4e. The plot shows that AgNPs yield was promoted by a low level of lactose and a high level of fructose. The effect of sucrose and lactose on the synthesis of AgNPs is plotted in Figure 4f. With a low level of lactose, initial sucrose caused maximum production of AgNPs.

### 3.2. UV–Visible Spectroscopy Analysis

**Figure 5:**
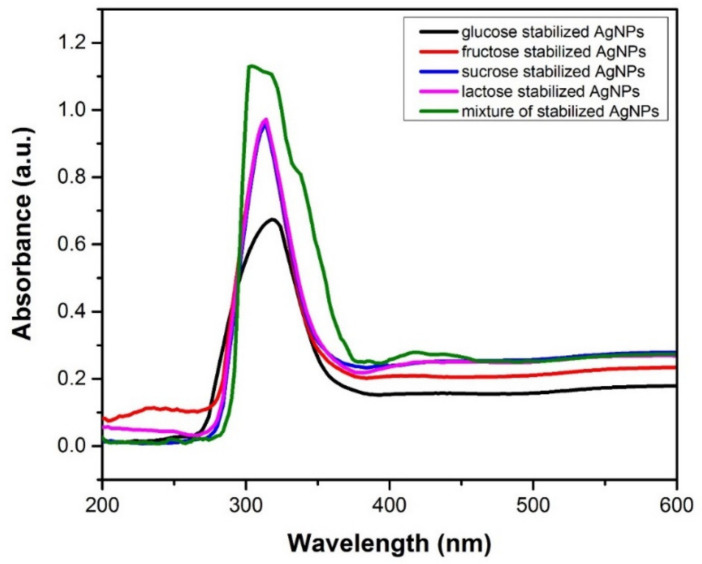
UV absorption spectra of individual and mixture of stabilized AgNPs.

UV–visible spectra were produced to analyze the optical domains of the prepared AgNPs. UV–visible spectroscopy was used to monitor the formation of AgNPs produced by the oxidation-reduction of a constant amount of AgNO_3_ mixed with pure stabilizers individually and their mixtures in water. During the plasma exposure of the solution of AgNO_3_ and stabilizers, the color of the solution started to change over time. The transparent solution turned to black by confirming the formation of AgNPs. The Ag^+^ ions reduced to AgNPs by absorbing the plasma generated species. UV–visible spectroscopy was used to confirm the formation of nanoparticles. A shown in Figure 5, a broad absorption band was observed around 200–600 nm. The maximum absorption was observed around 300–330 nm, corresponding to a typical plasmon resonance band of AgNPs. It is worth noting that the addition of pure stabilizers in the AgNO_3_ solution (glucose, fructose, sucrose and lactose) results in absorption bands at nearly the same wavelengths and the absorption peak intensity decreases with an increase in pure stabilizing content, which is an indication of an increase in the production of AgNPs.

The published literature suggests that AgNPs, produced with AgNO_3_ salt in the presence of different stabilizing agents, have UV peaks in the wavelength range of 300 nm [40,41]. The SPR peak shifts toward the shorter wavelength with a decrease in the absorbance due to the increased destabilization of the nanoparticles. The spontaneous growth of nanoparticles on plasma exposure is referred to the direct redox of Ag^+^ ions and oxidation of hydroxyl groups of the stabilizers [42]. It is also reported that the formation of AgNPs is mainly due to the redox of Ag^+^ ions by the irradiation effect and forming of hydrogen bonds with OH and –NH_2_ groups of the stabilizer [43]. To optimize the effect of pure and mixed stabilizers in AgNPs formation, simplex centroid (SCD) was utilized. The intensity of surface plasmon peak absorption increased from 0.1 to 1.6 in SCD results. These findings indicate that a stabilizer mixture acts as an interface between the reduction process and the formation of AgNPs. Free electrons exist in metal NPs, and their combined vibration in resonance with the light wave produces an SPR absorption band as elaborated by Ansar et al. [44]. Only one SPR band is expected in case of spherical NPs, while in the case of anisotropic shape, two or more SPR bands can be produced with the absorption peak related to the particle size. As particle size increases, the SPR peak of AgNPs in aqueous solution shifts towards longer wavelengths. The shape and sizes of AgNPs are directly affected by the synthesis parameters such as stabilizing agents, surface adsorbed particles and the dielectric constant of the medium [45,46].

### 3.3. FT-IR Study

**Figure 6:**
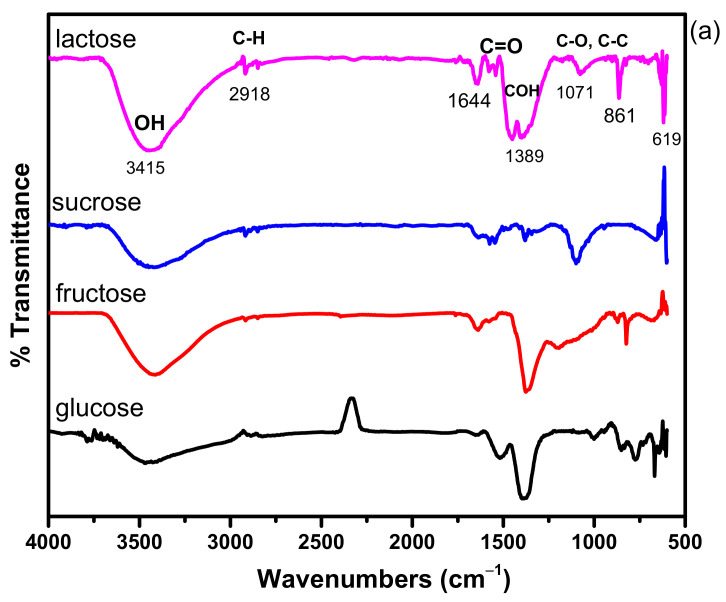

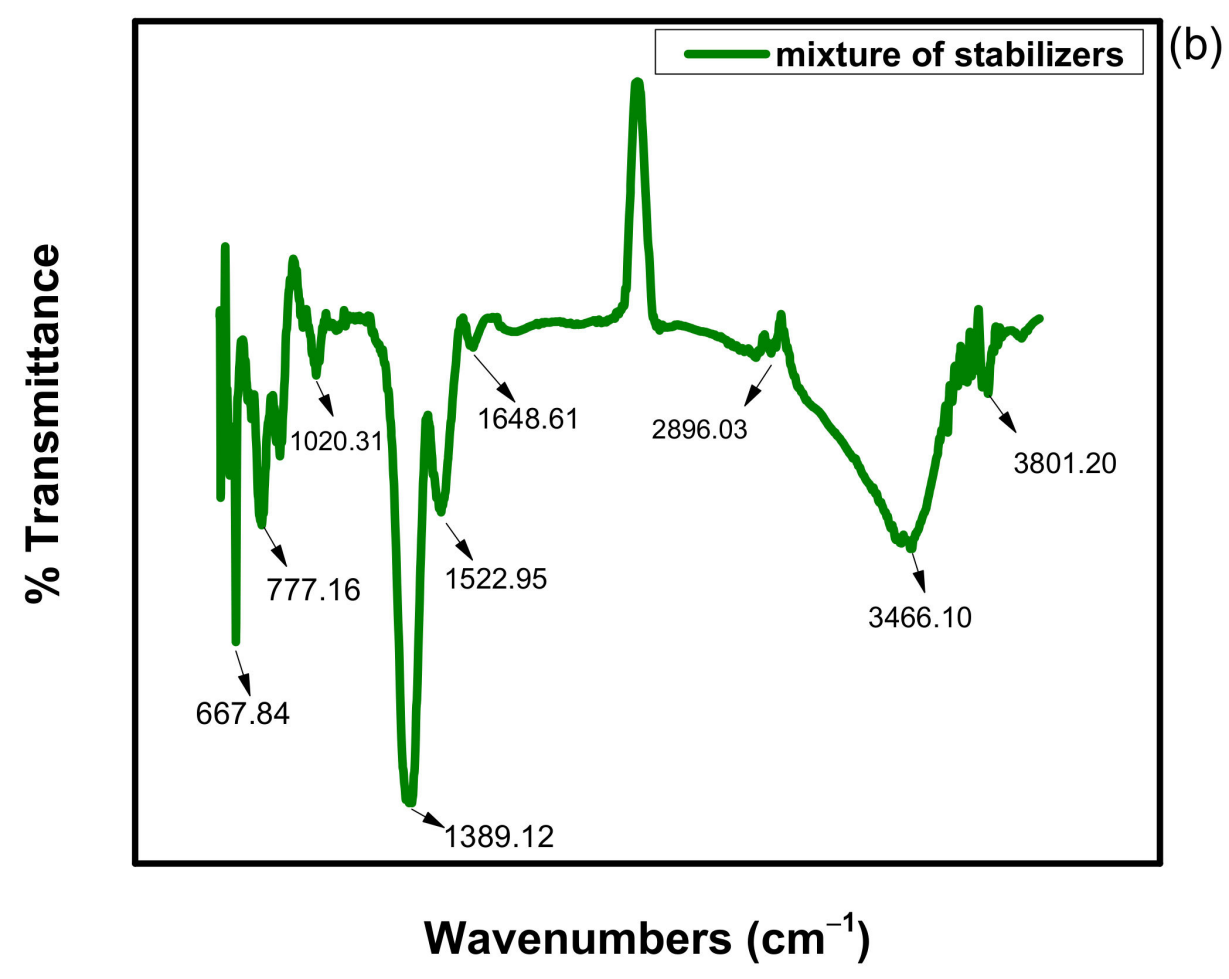
(**a**) FTIR spectra of AgNPs synthesized using pure stabilizers and (**b**) an optimized mixture of stabilizers.

FT-IR analysis confirmed the presence of various functional groups, which are essential to study the surface chemical state of the synthesized AgNPs and the possible interactions between Ag and stabilizing agents. FT-IR spectra of (a) glucose stabilized (run 3), (b) fructose stabilized (run 24), (c) sucrose stabilized (run 1), (d) lactose stabilized (run seven) and (e) mixture of four stabilizers (run 16) under optimized conditions (60% AgNO_3_, 40% stabilizing agent) are shown in Figure 6a,b. FT-IR spectra exhibit a broader spectrum with few peaks of sugar-capped AgNPs. The sharp spectral peak at 2896 cm^−1^ was attributed to C-H single-bonded alkane group. The band stretching observed near 1020.31 cm^−1^ may be attributed to C-N stretching of amine group. Similarly, the peak appeared at 777 cm^−1^ was attributed to a combination band stretching of CCO and CCH. The absorption peak of Ag/saccharides, observed at 1648 cm^−1^, was attributed at C=O. Meshram et al. [47] detected the same peak at 1649 cm^−1^, which shows a relationship between AgNPs and carbonyl group in saccharides. This could be due to the carbonyl group’s oxygen atoms, which allow these saccharides to bind to the surfaces of Ag particles. The data represented the interaction of the corresponding band with AgNPs (Figure 6a,b). UV–Vis analysis confirms a peak at 302 nm (Figure 5), which attributed to the electronic excitations of amine and carbonyl group in saccharides. The broad band in the range of 300–350 nm is assigned to the combination of the amino acids, which are incorporated as a part of glycoprotein with the help of saccharides. The amino acids are covalently attached to many different proteins. The UV peak of sugar stabilized AgNP complexes in literature is reported at about 300 nm [40,41,48,49,50]. The nanoparticles interact with stabilizing agents through electrostatic interaction with free amine groups or asparagine residues in proteins.

### 3.4. XRD Analysis

**Figure 7:**
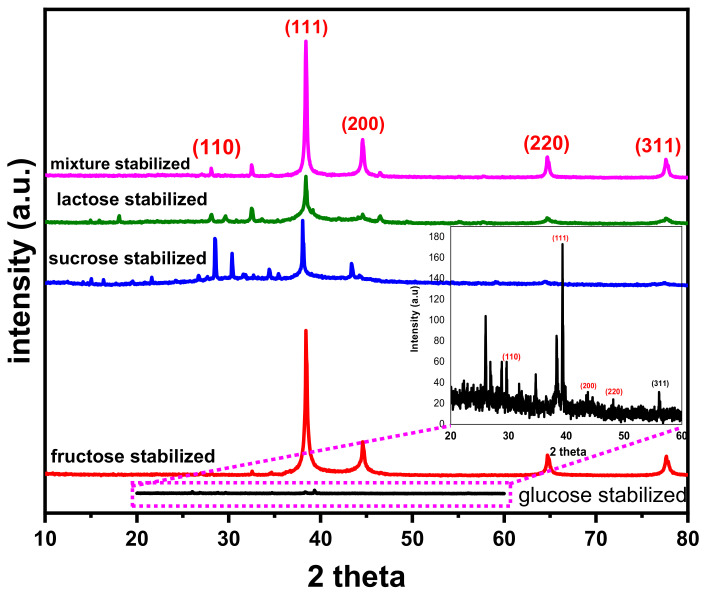
XRD spectra of AgNPs stabilized with pure and mixture of stabilizers.

The crystalline phase of the synthesized AgNPs was estimated using XRD analysis. The obtained XRD spectra of AgNPs ranged from 20° to 80°, as shown in Figure 7. X-ray diffraction patterns of synthesized AgNPs using various saccharides exhibited four distinct diffraction peaks at 38.41°, 44.63°, 64.75° and 77.83°, corresponding to (111), (200), (220) and (311) planes, respectively. The sharp peak at 38.41° revealed the face centered cubic (FCC) structure of Ag, which corresponds to the plane (111). The crystallite size calculations were conducted using Scherrer equation and results are summarized in Table 4. The crystallite size was smaller when pure stabilizers were used as stabilizing agent. The diffraction peaks of AgNPs for mixture of stabilizers are slightly smoother and wider than for individual stabilizers. The average particle size of AgNPs varied from 5–15 nm, depending on the stabilizer type [51]. It shows that stabilizing agents play a decisive role in controlling the growth of AgNPs. The obtained XRD results were in good matching with the structural analysis of AgNPs performed by Hassanien and Khatoon [52].

### 3.5. SEM and EDX Analysis

**Figure 8:**
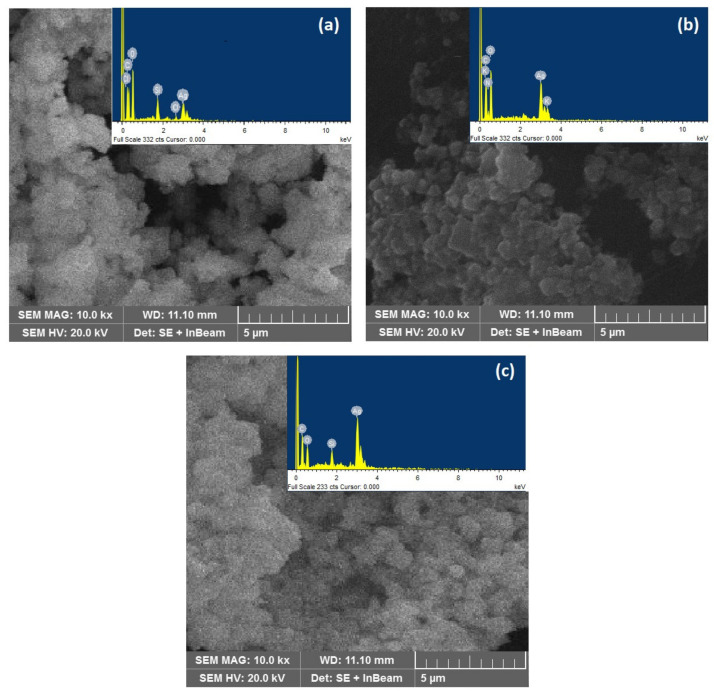
SEM micrographs and EDX spectra of (**a**) glucose stabilized AgNPs (run 3), (**b**) fructose stabilized AgNPs (run 24) and (**c**) AgNPs stabilized with a mixture of stabilizers (run 16).

The surface morphology and elemental analysis of the prepared AgNPs were performed by the scanning electron microscopy–energy dispersive X-ray (SEM-EDX) technique. The SEM images show that sugar stabilizers improved the colloid stability, agglomeration prevention and provided better control of particle size of the prepared AgNPs. In the absence of the stabilizers, AgNPs showed a poly-dispersed size distribution having irregular dendritic clusters [53,54]. Mono- and poly-saccharides are soft stabilizing agents that are crucial for the synthesis of AgNPs. The concentration of stabilizing agents has been varied up to 40% to obtain the optimum sugar concentration. Figure 8 shows SEM images of AgNPs produced with individual and mixed saccharides. The prepared AgNPs exhibited a nearly spherical shape along with some agglomerates. From XRD, the mixture of four stabilizers increases the average sizes of AgNPs, which may occur due to the increase in reaction rate resulting in more nuclei production and accelerated lateral growth of nanoparticles in short periods of time. There was also some aggregation and the majority of our analysis results revealed distinct spherical particles. Wang et al. [55] reported the formation of aggregates when low concentrations of glucose was used in the synthesis of AgNPs. On reaction with the stabilizing sugars in under 30 min plasma exposure, the colorless AgNO_3_ solution turned black, indicating the reduction of Ag^+^ to Ag◦. EDX revealed 66.67% of silver content in the mixture stabilized run 16 under the set conditions, as shown in Table 5.

Figure 8 also shows EDX spectrum of the synthesized AgNPs. The solid absorption signal was recorded around 3 keV, which clearly shows the existence of Ag content in the prepared AgNPs. EDX spectrum also confirmed the presence of C, O, Si, K, Cl and Ag absorbance peaks. The elemental signals of Ca and Na obtained in EDX spectrum may appear due to the sugar solution and/or likely to be produced by X-ray emissions from the glass substrate utilized during EDX analysis, as revealed earlier by Nangia et al. [40]. FT-IR and EDX studies revealed that AgNPs can be functionalized by using different stabilizers/saccharides during the synthesis process. AgNPs were produced from a solution of silver nitrate combined with saccharides exposed to a plasma jet. Since the graphite rod was used as a plasma anode, which was in direct contact with the solution, the possible reason of carbon in the product was arcing and sputtering of the graphite during plasma exposure. The presence of proteins and glycoproteins in the product is attributed to the used saccharides.

### 3.6. Antibacterial Activity

**Figure 9:**
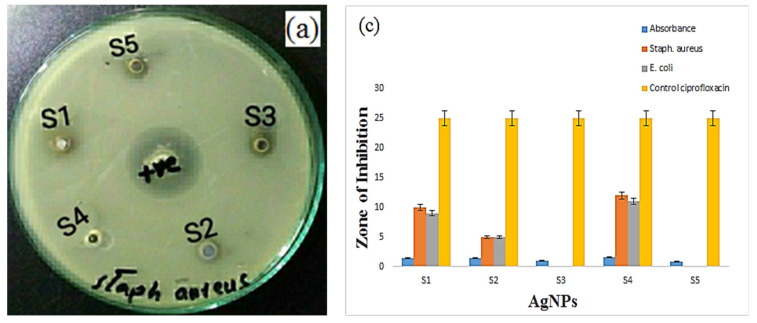

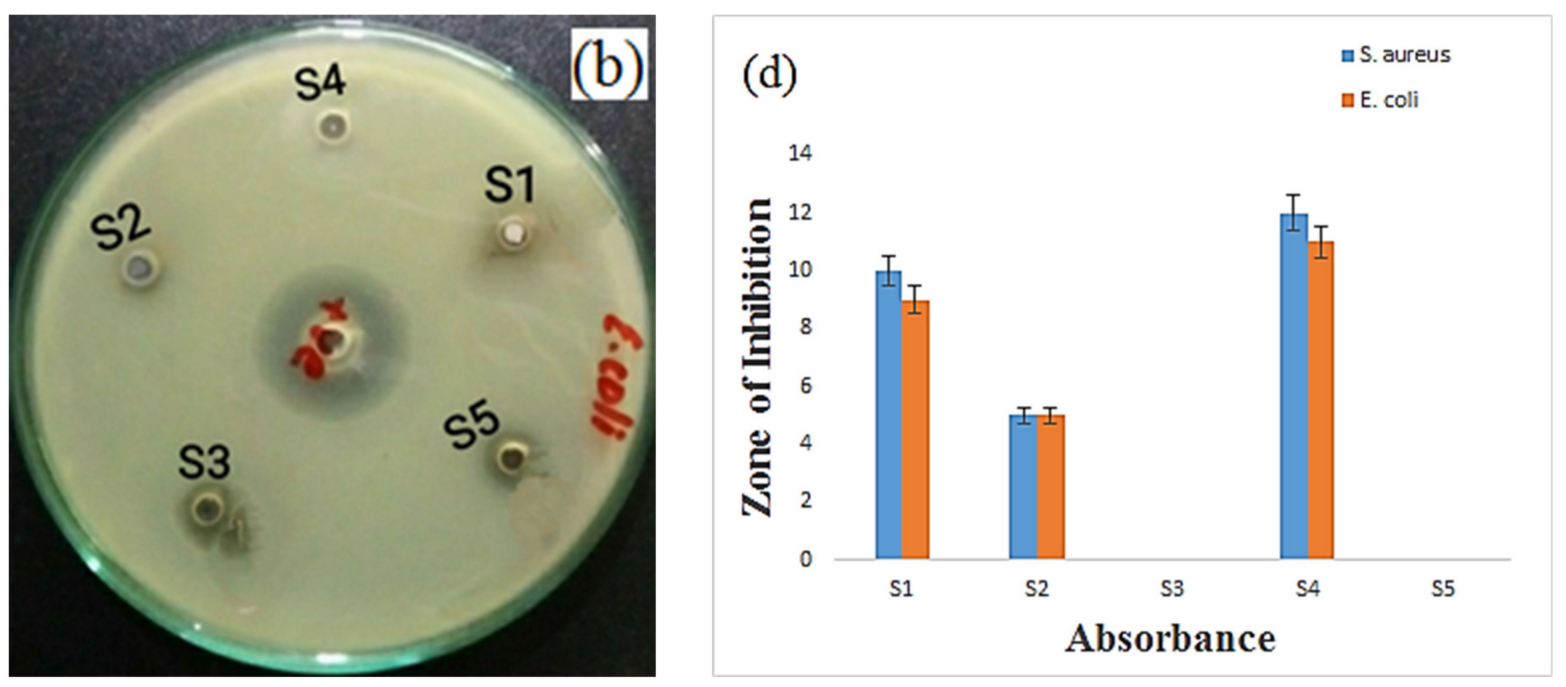
Photographs of inhibition zones of AgNPs with different stabilizing agents (**a**) Staph aureus and (**b**) *E. coli* and (**c**) comparative graph for zone of inhibition with absorbance and control; (**d**) representative graph of antibacterial activity.

The majority of *E. coli* strains cause mild diarrhea in humans. However, some of *E. coli* strains cause bloody diarrhea, severe stomach cramps and vomiting. These gram-negative strains are found in contaminated water and food polluted by animal waste, human waste, industrial effluents and the food processing industry. Some of the *E. coli* strains are found in municipal water supply as well. Some people also get infected with such strains after swimming in contaminated pools or lakes. On the other hand, *Staph aureus* is a gram-positive strain, which causes inflammatory diseases in human. It is commonly found in the environment and is spread to humans via aerosol and air droplets when an infected person sneezes or coughs. The antimicrobial activity of optimized AgNPs (run number 1, 3, 7, 16 and 24) against *Staph aureus* and *E. coli* strains was evaluated by using the shake flask method. Phosphate buffered saline (PBS) having a pH value of 7.2 was used as a standard against all the five bacterial strains. About 0.2 mL of bacterial inoculums were added to the control and test samples. The samples were saturated in 20 mL of nutrient broth and shaken at 110 rpm under a constant temperature of 37 °C. After 24 h of incubation period at 37 °C, the antibacterial activity of the control and AgNPs was determined by measuring the diameter of the inhibition in the individual samples. Ciprofloxacin was used as a control. The nanoparticles showed an inhibitory effect on gram-positive and gram-negative bacteria. Smaller nanoparticles showed better antibactericidal impact than larger nanoparticles due to their larger reactive area. It is suggested that silver ions with a positive charge form an electrostatic attraction with a negatively charged microbial membrane. Typical images of the inhibition zone of AgNPs with the combination of different stabilizing agents are shown in Figure 9. The results of zone inhabitation of the samples S1 (run 1), S2 (run 3), S3 (run 7), S4 (run 16), S5 (run 24) are shown in Table 6. The zone inhibition of AgNPs with different stabilizing agents against *E. coli* was measured about 9 mm, 5 mm 2 mm, 11 mm and 4 mm, respectively. The zone inhibition of AgNPs with different stabilizing agents against *Staph aureus* was measured about 10 mm, 5 mm, 4 mm, 12 mm and 5 mm, respectively. The zone inhibition to be increased with an increase in absorbance, as observed in Figure 9c,d. The effective zone of the combination of stabilizing agents was measured about 11 mm and 12 mm against both bacterial colonies, respectively. This shows that the antibacterial activity of AgNPs depends on the type of stabilizing agents. The zone of inhibition less than 5 mm implies moderate resistance, while a zone of inhibition higher than 10 mm suggests significant resistance, as suggested by Xuan et al. [56].

### 3.7. Future Prospects

The liquid–plasma interaction is an effective way of producing nanomaterials. It is simple, easy to hand and a low-cost approach, which do not contribute much to environmental pollution. However, many aspects of this approach need to be investigated further to have better control over structural growth and yield of the nanomaterials. For example, there are very few studies on statistical optimization of the process parameters for controlled production of nanomaterials. The effect of sugar stabilizers on particle size and UV absorbance was statistically optimized in this work. Future research should concentrate on different types of stabilizers rather than a single class of stabilizers. The plasma–liquid interaction parameters, such as gas type, plasma jet design, input power gas flowrate, etc., should also be optimized statistically using different statistical models. We performed synthesis experiments in an open atmosphere, which might be energy intensive since high voltage is required to sustain the plasma jet under atmospheric pressure. Similar, experiments can be conducted under low pressure conditions in a vacuum tight chamber to lower the required voltage.

## 4. Conclusions

A facile and rapid liquid–plasma interaction technique was used to synthesize AgNPs with mono- and/or poly-saccharides as stabilizing agents. The effect of pure glucose, fructose, sucrose and lactose and their combinations on the structure and yield of AgNPs was investigated by SCD. A combination of selected surface-active compounds produced particles of the desired size and shape for the optical and antibacterial properties. A linear model with R^2^ = 0.9657 showed better efficiency of SSD in the synthesis of AgNPs through the LPI technique. The combined and individual effect of stabilizers on the synthesis of AgNPs was significant when *p* < 0.05. The present method for AgNPs synthesis is eco-friendly and pollutant-free as it requires low temperature, non-toxic reagents, surfactants and/or organic solvents for NPs synthesis. The nearly spherical particles with a high yield having an average size of 15 nm were obtained under optimal conditions using a 40% mixture of stabilizers. The overall UV absorbance of AgNPs was obtained around 302 nm. The FT-IR study showed that the mixture of saccharides might have played an important role in the stabilization of AgNPs through the coating of a carbohydrates moiety on AgNPs. The SEM and UV–vis spectroscopy analysis showed that in the presence of sugar stabilizers, small and nearly spherical particles with an average size of 9–15 nm were produced using the LPI method. The antibacterial activity of these nanoparticles was also tested against *Staph aureus* and *E. coli* strains. The zone inhibition of AgNPs with different stabilizing agents against *E. coli* varied from 2 nm to 9 mm. Similarly, the zone inhibition against *Staph aureus* varied from 4 nm to 10 mm.

## Figures and Tables

**Table 1:** SCD with four components (A, B, C, D) with none process variable.

	Stabilizing/Capping Agents 5-Levels				Absorbance at 302 nm Experimental	Response
Run	Glucose (A)	Fructose (B)	Sucrose (C)	Lactose (D)		Predicted
1	0	2	0	0	1.488	1.329
2	−2	0	0	0	0.570	0.495
3	0	−2	0	0	1.438	1.531
4	2	0	0	0	0.540	0.501
5	0	0	2	0	0.285	0.212
6	0	0	0	0	0.279	0.289
7	0	0	−2	0	1.028	1.085
8	0	0	0	0	0.640	0.501
9	0	0	0	−2	0.580	0.555
10	0	0	0	2	0.620	1.533
11	1	1	−1	−1	0.011	−0.124
12	1	−1	1	−1	0.570	0.581
13	1	1	−1	1	0.670	0.688
14	1	1	1	−1	0.590	0.600
15	0	0	0	0	0.350	0.352
16	−1	−1	−1	−1	1.626	1.534
17	1	−1	−1	1	0.500	0.501
18	0	0	0	0	0.270	0.280
19	−1	1	1	1	0.493	0.491
20	−1	−1	1	1	0.037	1.122
21	1	−1	1	1	0.83	0.811
22	−1	−1	−1	1	0.570	0.495
23	−1	1	−1	1	0.395	0.300
24	−1	1	−1	−1	0.871	0.844
25	1	1	1	1	0.395	0.394
26	−1	1	1	−1	0.300	0.305
27	0	0	0	0	0.041	0.042
28	0	0	0	0	0.011	−0.124
29	1	−1	−1	−1	0.041	−0.020
30	1	−1	1	−1	0.037	0.004

**Table 2:** Fit Statistics of sugar-stabilized AgNPs using SCD design.

Variables	Coefficients	Main Effect	*t*-Stat	*p*-Value	C.I (%)
Intercept	0.395	0.790	8.816	0.000	100.000
Glucose (A)	−0.028	−0.056	−1.260	0.032	96.754
Fructose (B)	0.247	0.494	11.024	0.000	100.000
Sucrose (C)	0.330	0.660	14.80	0.000	100.000
Lactose (D)	−0.053	−0.106	−2.367	0.228	77.279
AB	−0.095	−0.190	−3.469	0.003	99.657
AC	−0.013	−0.025	−0.457	0.564	34.639
AD	0.091	0.182	3.324	0.005	99.538
BC	−0.024	−0.047	−0.959	0.404	59.603
BD	−0.026	−0.053	−0.859	0.343	65.724
CD	−0.054	−0.108	−1.965	0.068	93.179
AA = A^2^	0.011	0.022	0.525	0.000	99.960
BB = B^2^	0.095	0.190	4.534	0.706	39.235
CC = C^2^	0.199	0.238	5.694	0.000	99.996
DD = D^2^	0.000	0.000	0.000	1.000	0.039

**Table 3:** Statistical values of ANOVA for optimization of AgNPs using SCD.

Source	Degree of Freedom	Sum of Squares	Mean Sum of Squares	Level of Significance (*p*-Value)	F-Value
Regression	14	5.094	0.364	0.000	30.201
Residual	15	0.081	0.013		
Total	29	5.275			

**Table 4:** Crystallite size of AgNPs stabilized with pure and mixed stabilizers.

Assays	Sample Description (AgNPs)	2 Theta (°)	Particle Size (nm)
Run 1	Sucrose stabilized	43.85	12 nm
Run 3	Glucose stabilized	24.934	10 nm
Run 7	Lactose stabilized	22.983	9.45 nm
Run 16	Mixture of four stabilizers	48.66	15 nm
Run 24	Fructose stabilized	34.03	11 nm

**Table 5:** Elemental analysis of prepared AgNPs (run 3, run 24, run 1, run 7 and run 16) using five spectrums focused at three distinct elements.

Experimental Run	Silver (Ag)		Oxygen (O)		Carbon (C)	
	Weight (%)	Atomic (%)	Weight (%)	Atomic (%)	Weight (%)	Atomic (%)
Run 3	33.34	6.67	39.00	52.57	19.27	34.60
Run 24	43.74	9.79	29.74	44.87	14.89	29.92
Run 1	45.40	10.63	29.18	46.05	17.88	37.58
Run 7	36.39	6.78	31.66	39.77	31.95	53.45
Run 16	69.81	24.45	16.48	38.91	10.11	31.80

**Table 6:** Inhibitory effect of optimized AgNPs growth with different stabilizing agents.

Sample	Runs	Absorbance	*Staph aureus*	*E. coli*	Control Ciprofloxacin
S1	1	1.488	10 mm	9 mm	25 mm
S2	3	1.438	5 mm	5 mm	25 mm
S3	7	1.028	4 mm	2 mm	25 mm
S4	16	1.626	12 mm	11 mm	25 mm
S5	24	0.871	5 mm	4 mm	25 mm

## Data Availability

Data supporting reported results is available and provided at reasonable request.

## References

[1] Behbudi G. (2021). Effect of silver nanoparticles disinfectant on covid-19. Adv. Appl. NanoBio-Technol..

[2] Boateng J., Catanzano O. (2020). Silver and silver nanoparticle-based antimicrobial dressings. Ther. Dress. Wound Heal. Appl..

[3] Eid A.M., Fouda A., Niedbała G., Hassan S.E.-D., Salem S.S., Abdo A.M., Hetta H.F., Shaheen T.I. (2020). Endophytic Streptomyces laurentii mediated green synthesis of Ag-NPs with antibacterial and anticancer properties for developing functional textile fabric properties. Antibiotics.

[4] LewisOscar F., Nithya C., Vismaya S., Arunkumar M., Pugazhendhi A., Nguyen-Tri P., Alharbi S.A., Alharbi N.S., Thajuddin N. (2021). In vitro analysis of green fabricated silver nanoparticles (AgNPs) against Pseudomonas aeruginosa PA14 biofilm formation, their application on urinary catheter. Prog. Org. Coat..

[5] Hajji S., Salem R.B.S.-B., Hamdi M., Jellouli K., Ayadi W., Nasri M., Boufi S. (2017). Nanocomposite films based on chitosan–poly (vinyl alcohol) and silver nanoparticles with high antibacterial and antioxidant activities. Process. Saf. Environ. Prot..

[6] Wu S., Tan N., Lan D., Au C.-T., Yi B. (2020). Hydrothermal Fabricated Ag Nanoparticles-decorated Reduced Graphene Oxide Composite for H2O2 Electrochemical Detection. Int. J. Electrochem. Sci..

[7] Ayyaz M., Shukrullah S., Naz M.Y., AbdEl-Salam N.M., Ibrahim K.A., Mohamed H.F. (2021). Microwave Plasma Assisted Sol-Gel Synthesis of TiO2 Photocatalyst for DC Plasma Jet Driven Degradation of Methylene Blue. ChemistrySelect.

[8] Korkmaz N., Akar K.B., İmamoğlu R., Kısa D., Karadağ A. (2021). Synthesis of silver nanowires in a two-phase system for biological applications. Appl. Organomet. Chem..

[9] Nakamura S., Ando N., Sato M., Ishihara M. (2020). Ultraviolet Irradiation Enhances the Microbicidal Activity of Silver Nanoparticles by Hydroxyl Radicals. Int. J. Mol. Sci..

[10] Tran L.T., Tran H.V., Dang H.T.M., Huynh C.D., Mai T.A. (2020). Silver nanoparticles decorated polyaniline nanowires-based electrochemical DNA sensor: Two-step electrochemical synthesis. J. Electrochem. Soc..

[11] Erol K., Bolat M., Tatar D., Nigiz C., Köse D.A. (2019). Synthesis, characterization and antibacterial application of silver nanoparticle embedded composite cryogels. J. Mol. Struct..

[12] Ahmad I., Shukrullah S., Ahmad M., Ahmed E., Naz M.Y., Akhtar M.S., Khalid N., Hussain A., Hussain I. (2020). Effect of Al doping on the photocatalytic activity of ZnO nanoparticles decorated on CNTs and graphene: Solvothermal synthesis and study of experimental parameters. Mater. Sci. Semicond. Process..

[13] Salem J.K., Draz M.A. (2020). Synthesis and application of silver nanorods for the colorimetric detection of sulfate in water. Inorg. Chem. Commun..

[14] Ayyaz M., Naz M., Shukrullah S., Altaf N. (2021). Microwave Plasma Assisted Sol-gel Technique for Synthesis of TiO2 Nanoparticles: A Recent Study. Newest Updates Phys. Sci. Res..

[15] Hemmati S., Harris M.T., Barkey D.P. (2020). Polyol Silver Nanowire Synthesis and the Outlook for a Green Process. J. Nanomater..

[16] Saratale R.G., Saratale G.D., Cho S.-K., Ghodake G., Kadam A., Kumar S., Mulla S.I., Kim D.S., Jeon B.H., Chang J.S. (2019). Phyto-fabrication of silver nanoparticles by Acacia nilotica leaves: Investigating their antineoplastic, free radical scavenging potential and application in H2O2 sensing. J. Taiwan Inst. Chem. Eng..

[17] Saratale R.G., Shin H.-S., Kumar G., Benelli G., Ghodake G.S., Jiang Y.Y., Kim D.S., Saratale G.D. (2017). Exploiting fruit byproducts for eco-friendly nanosynthesis: Citrus × clementina peel extract mediated fabrication of silver nanoparticles with high efficacy against microbial pathogens and rat glial tumor C6 cells. Environ. Sci. Pollut. Res..

[18] Parham S., Kharazi A.Z., Bakhsheshi-Rad H.R., Ghayour H., Ismail A.F., Nur H., Berto F. (2020). Electrospun Nano-Fibers for Biomedical and Tissue Engineering Applications: A Comprehensive Review. Materials.

[19] Shukrullah S., Ayyaz M., Naz M.Y., Ibrahim K.A., AbdEl-Salam N.M., Mohamed H.F. (2021). Post-synthesis plasma processing and activation of TiO2 photocatalyst for the removal of synthetic dyes from industrial wastewater. Appl. Phys. A.

[20] Kumar S.V., Bafana A.P., Pawar P., Rahman A., Dahoumane S.A., Jeffryes C.S. (2018). High conversion synthesis of <10 nm starch-stabilized silver nanoparticles using microwave technology. Sci. Rep..

[21] Shukrullah S., Naz M.Y., Altaf N.U.H., Ali A. (2020). Effect of DC voltage on morphology and size distribution of silver nanorods synthesized through plasma-liquid interaction method. Mater. Today Proc..

[22] Kiran G.S., Selvin J., Manilal A., Sujith S. (2010). Biosurfactants as green stabilizers for the biological synthesis of nanoparticles. Crit. Rev. Biotechnol..

[23] BBrycki B., Szulc A., Babkova M. (2020). Synthesis of Silver Nanoparticles with Gemini Surfactants as Efficient Capping and Stabilizing Agents. Appl. Sci..

[24] Saravanakumar K., Hu X., Chelliah R., Oh D.-H., Kathiresan K., Wang M.-H. (2019). Biogenic silver nanoparticles-polyvinylpyrrolidone based glycerosomes coating to expand the shelf life of fresh-cut bell pepper (Capsicum annuum L. var. grossum (L.) Sendt). Postharvest Biol. Technol..

[25] Ayyaz M., Huda N.U., Rasool F., Sami-Ur-Rehman H., Mehmood A., Naz M.Y., Shukrullah S., Ghaffar A. (2020). Effects of pure and mixed stabilizers on opto-electrical properties and morphology of TiO2 nanoparticles synthesized by sol-gel method. IOP Conf. Series Mater. Sci. Eng..

[26] Hamouda R.A., El-Mongy M.A., Eid K.F. (2019). Comparative study between two red algae for biosynthesis silver nanoparticles capping by SDS: Insights of characterization and antibacterial activity. Microb. Pathog..

[27] Iqbal M., Zafar H., Mahmood A., Niazi M.B.K., Aslam M.W. (2020). Starch-Capped Silver Nanoparticles Impregnated into Propylamine-Substituted PVA Films with Improved Antibacterial and Mechanical Properties for Wound-Bandage Applications. Polymers.

[28] Squeo G., De Angelis D., Leardi R., Summo C., Caponio F. (2021). Background, Applications and Issues of the Experimental Designs for Mixture in the Food Sector. Foods.

[29] Nunes N.D.S., Carneiro L.L., De Menezes L.H.S., De Carvalho M.S., Pimentel A.B., Silva T.P., Pacheco C.S.V., Tavares I.M.D.C., Santos P.H., Das Chagas T.P. (2020). Simplex-Centroid Design and Artificial Neural Network-Genetic Algorithm for the Optimization of Exoglucanase Production by Penicillium Roqueforti ATCC 10110 Through Solid-State Fermentation Using a Blend of Agroindustrial Wastes. BioEnergy Res..

[30] Beg S. (2021). Mixture Designs and Their Applications in Pharmaceutical Product Development. Design of Experiments for Pharmaceutical Product Development.

[31] Zhai L., Lu Y., Chen D., Chen X., Liu L., Li C. (2021). Increase the rate of plasma-assisted synthesis of silver nanoparticles through additives. E3S Web Conf..

[32] Altaf N., Naz M., Shukrullah S., Bhatti H. (2021). Testing of photocatalytic potential of silver nanoparticles produced through nonthermal plasma reduction reaction and stabilized with saccharides. Main Group Chem. Preprint.

[33] Ibrahim S., Ahmad Z., Manzoor M.Z., Mujahid M., Faheem Z., Adnan A. (2021). Optimization for biogenic microbial synthesis of silver nanoparticles through response surface methodology, characterization, their antimicrobial, antioxidant, and catalytic potential. Sci. Rep..

[34] Azmi S.N.H., Al-Jassasi B.M.H., Al-Sawafi H.M.S., Al-Shukaili S.H.G., Rahman N., Nasir M. (2021). Optimization for synthesis of silver nanoparticles through response surface methodology using leaf extract of Boswellia sacra and its application in antimicrobial activity. Environ. Monit. Assess..

[35] Berkani M., Kadmi Y., Bouchareb M.K., Bouhelassa M., Bouzaza A. (2020). Combinatıon of a Box-Behnken design technique with response surface methodology for optimization of the photocatalytic mineralization of C.I. Basic Red 46 dye from aqueous solution. Arab. J. Chem..

[36] Mahmoud G.A.-E., Bashandy S.R. (2021). Nitrogen, Amino Acids, and Carbon as Control Factors of Riboflavin Production by Novosphingobium panipatense-SR3 (MT002778). Curr. Microbiol..

[37] Alkholief M. (2019). Optimization of Lecithin-Chitosan nanoparticles for simultaneous encapsulation of doxorubicin and piperine. J. Drug Deliv. Sci. Technol..

[38] Barabadi H., Honary S., Ebrahimi P., Alizadeh A., Naghibi F., Saravanan M. (2019). Optimization of myco-synthesized silver nanoparticles by response surface methodology employing Box-Behnken design. Inorg. Nano-Metal Chem..

[39] Sheng Z., Li J., Li Y. (2012). Optimization of ultrasonic-assisted extraction of phillyrin from Forsythia suspensa using response surface methodology. J. Med. Plants Res..

[40] Aydin I., Ertekin K., Oncuoglu S., Hizliates C.G. (2021). Manipulating spectral properties of the Hg (II) sensitive carbazole-oxadiazole derivative by silver nanoparticles: Two different sensing mechanisms for the same probe. Opt. Mater..

[41] Mirzaei S.Z., Lashgarian H.E., Karkhane M., Shahzamani K., Alhameedawi A.K., Marzban A. (2021). Bio-inspired silver selenide nano-chalcogens using aqueous extract of Melilotus officinalis with biological activities. Bioresour. Bioprocess..

[42] Kondeti V.S.S.K., Gangal U., Yatom S., Bruggeman P. (2017). Ag+ reduction and silver nanoparticle synthesis at the plasma–liquid interface by an RF driven atmospheric pressure plasma jet: Mechanisms and the effect of surfactant. J. Vac. Sci. Technol. A.

[43] Khan A., Khan R.A., Salmieri S., Le Tien C., Riedl B., Bouchard J., Chauve G., Tan V., Kamal M.R., Lacroix M. (2012). Mechanical and barrier properties of nanocrystalline cellulose reinforced chitosan based nanocomposite films. Carbohydr. Polym..

[44] Ansar S., Tabassum H., Aladwan N.S., Ali M.N., Almaarik B., AlMahrouqi S., Abudawood M., Banu N., Alsubki R. (2020). Eco friendly silver nanoparticles synthesis by Brassica oleracea and its antibacterial, anticancer and antioxidant properties. Sci. Rep..

[45] Burda C., Chen X., Narayanan R., El-Sayed M.A. (2005). Chemistry and Properties of Nanocrystals of Different Shapes. Chem. Rev..

[46] Lin L., Li X., Zhou J., Zou J., Lai J., Chen Z., Shen J., Xu H. (2021). Plasma-aided green and controllable synthesis of silver nanoparticles and their compounding with gemini surfactant. J. Taiwan Inst. Chem. Eng..

[47] Meshram S.M., Bonde S.R., Gupta I.R., Gade A.K., Rai M.K. (2013). Green synthesis of silver nanoparticles using white sugar. IET Nanobiotechnol..

[48] Wahab S., Khan T., Adil M., Khan A. (2021). Mechanistic aspects of plant-based silver nanoparticles against multi-drug resistant bacteria. Heliyon.

[49] Abdelsattar A., Nofal R., Makky S., Safwat A., Taha A., El-Shibiny A. (2021). The Synergistic Effect of Biosynthesized Silver Nanoparticles and Phage ZCSE2 as a Novel Approach to Combat Multidrug-Resistant Salmonella enterica. Antibiotics.

[50] Ghramh H.A., Khan K.A., Ibrahim E.H., Ansari M.J. (2019). Biogenic synthesis of silver nanoparticles using propolis extract, their characterization, and biological activities. Sci. Adv. Mater..

[51] Sahoo P.K., Kamal S.S.K., Kumar T.J., Sreedhar B., Singh A.K., Srivastava S.K. (2009). Synthesis of Silver Nanoparticles using Facile Wet Chemical Route. Def. Sci. J..

[52] Hassanien A.S., Khatoon U.T. (2018). Synthesis and characterization of stable silver nanoparticles, Ag-NPs: Discussion on the applications of Ag-NPs as antimicrobial agents. Phys. B Condens. Matter.

[53] Ashraf S., Abbasi A.Z., Pfeiffer C., Hussain S.Z., Khalid Z.M., Gil P.R., Parak W.J., Hussain I. (2013). Protein-mediated synthesis, pH-induced reversible agglomeration, toxicity and cellular interaction of silver nanoparticles. Colloids Surf. B Biointerfaces.

[54] Bezza F.A., Tichapondwa S.M., Chirwa E.M. (2020). Synthesis of biosurfactant stabilized silver nanoparticles, characterization and their potential application for bactericidal purposes. J. Hazard. Mater..

[55] Wang J., Jiu J., Zhang S., Sugahara T., Nagao S., Suganuma K., He P. (2018). The comprehensive effects of visible light irradiation on silver nanowire transparent electrode. Nanotechnology.

[56] Devanesan S., AlSalhi M.S. (2021). Green Synthesis of Silver Nanoparticles Using the Flower Extract of Abelmoschus esculentus for Cytotoxicity and Antimicrobial Studies. Int. J. Nanomed..

